# Identification and verification of autophagy-related gene signatures and their association with immune infiltration and drug responsiveness in epilepsy

**DOI:** 10.3389/fneur.2024.1503632

**Published:** 2025-01-22

**Authors:** Han-han He, Xiao-ge Zhang, Fen-fang Chen

**Affiliations:** ^1^Department of Pediatrics, Northwest Women’s and Children’s Hospital, Xi’an, China; ^2^Department of Pediatrics, The Second Affiliated Hospital of University of South China, Hengyang, China

**Keywords:** epilepsy, autophagy, immune infiltration, anti-epileptic drug responsiveness, ITPR3

## Abstract

**Background:**

Epilepsy, a common neurological disorder, is characterized by susceptibility to recurrent seizures. Increasing evidence suggests that autophagy plays a crucial role in the initiation and progression of epilepsy. However, the precise mechanisms by which autophagy deficiencies involved in epileptogenesis are still not fully understood.

**Methods:**

Two datasets of epilepsy (GSE143272 and GSE256068) were downloaded from the Gene Expression Omnibus (GEO) database. Differential expression genes (DEGs) analysis and weighted gene co-expression network analysis (WGCNA) were employed to screen for autophagy related differential expression genes (ARDEGs) in GSE143272 database. Subsequently, protein–protein interaction, transcription factors and miRNAs networks were constructed. Additionally, the functional enrichment analysis of Gene Ontology (GO) and Kyoto Encyclopedia of Genes and Genomes (KEGG) were applied. The hub ARDEGs were identified through CytoHubba, followed by the LASSO analysis. The Immune Cell Abundance Identifier (ImmuCellAI) was used to estimate peripheral immune cells abundance of epilepsy. Furthermore, the expression level of hub ARDEGs were detected in patients treated with different epilepsy monotherapies to explore the role of autophagy in the responsiveness of antiepileptic drug therapy. Finally, the expression level of hub ARDEGs were further validated in hippocampus of GSE256068 to enhance the reliability of the results.

**Results:**

Twenty ARDEGs in epilepsy were screened out by integrating DEGs and WGCNA analysis. KEGG enrichment analysis showed that the ARDEGs in epilepsy were not only involved in the autophagy, but also apoptosis, the NOD-like receptor signaling pathway, the neurotrophin signaling pathway, etc. Four hub ARDEGs (PIK3R1, TRIM21, TRIM22, and ITPR3) were screened through integrating CytoHubba plug and LASSO analysis. The immune infiltration analysis showed that there was a significantly increased abundance of macrophages and a decreased abundance of CD4 and CD8 T cells, including Tr1, nTreg, Tfh, CD8 naïve, cytotoxic T cells and effector memory T cells in the epilepsy group. Furthermore, the hub ARDEGs were significantly correlated with the abundance of differential immune cells. In expression level validation and anti-epileptic drug responsiveness analysis, PIK3R1 and ITPR3 had significant differences in the hippocampus of patients with epilepsy. PIK3R1 expression level was found to be related with carbamazepine resistance.

**Conclusion:**

This study elucidated the autophagy-related gene signatures in epilepsy and clarified their association with immune infiltration and anti-epileptic drug responsiveness, providing a novel target for future therapeutic interventions and disease markers in epilepsy.

## 1 Introduction

Epilepsy is a type of highly heterogeneous neurological afflict individuals of all ages (1, 2). Its typical clinical manifestation is a long lasting predisposition to recurrently generate unprovoked seizures (3). According to the latest statistics, there are currently about 51 million active epilepsy patients and 4.9 million new-onset epilepsy patients worldwide (1). Regarding epilepsy treatment, anti-seizure medications are the main approach for the management of epilepsy (1). However, despite the fact that there are currently more than 25 anti-seizure medications available worldwide, a third of people with epilepsy fail to control their seizures (4). Therefore, epilepsy not only seriously impacts the quality of life of patients, but also brings a huge economic burden to the family and even the entire society. In 2017, the International League Against Epilepsy (ILAE) divided epilepsy into six categories, including genetic etiology, infectious etiology, metabolic etiology, structural etiology, immune etiology, and unknown etiology, providing a framework for both clinical practice and basic research (5). Numerous studies on the epileptogenesis process have indicated that the disruption in the balance between excitatory and inhibitory neurotransmission within the central nervous system (CNS) is the primary cause of abnormal neuron activity in the cerebral cortex (6). Nevertheless, the precise mechanism underlying epileptogenesis remains ambiguous and demands further clarification.

Autophagy is a cellular process which cells regulate their own functions and can be triggered by various signals, such as oxidative stress, nutrient deficiency, and the blockade of the mammalian target of rapamycin (mTOR) (7). Three types of autophagy that are mechanistically distinct are encompassed: macroautophagy, microautophagy and chaperone-mediated autophagy (CMA) (8). Autophagy is indispensable for maintaining cellular homeostasis by degrading toxins, pathogens, misfolded proteins, and damaged organelles (9). Mounting evidence indicates that autophagy plays a crucial role in the onset and progression of neurodegenerative diseases, including epilepsy (10). Recent studies have demonstrated a substantial connection between epilepsy and autophagy, as pathological alterations during epilepsy have been observed to be influenced by autophagy. This encompasses changes in imbalances of neuronal excitability and inhibition, synaptic structure and function, and abnormal connections within neural circuits (11, 12). Nevertheless, the precise mechanisms by which autophagy deficiencies are involved in epileptogenesis remain incompletely understood.

In this study, we endeavored to uncover the autophagy related gene (ARDEGs) signatures in epilepsy and explore their association with immune infiltration and resistance to antiepileptic drugs. First of all, differential expression genes (DEGs) analysis and weighted gene co-expression network analysis (WGCNA) were employed to screen for autophagy related differential expression genes (ARDEGs) in epilepsy. Subsequently, protein–protein interaction (PPI) network, transcription factors (TF) and miRNA-gene regulatory network were constructed. To investigate the probable molecular mechanism, the functional enrichment analysis of Gene Ontology (GO) and the Kyoto Encyclopedia of Genes and Genomes (KEGG) were applied. Furthermore, the hub genes were identified through various algorithm from CytoHubba, followed by the least absolute shrinkage and selection operator (LASSO) logistic regression analysis. The Immune Cell Abundance Identifier (ImmuCellAI) tool was utilized to predict the proportion of immune cells in epilepsy and the correlation between the differential abundance of immune cells and the hub ARDEGs expression level was analyzed. Finally, the hub ARDEGs were validated in independent epileptic datasets and anti-epileptic drug responsiveness related data to enhance the reliability of the experimental results. This study will furnish a new foundation for in-depth comprehension of the pathogenesis of autophagy and exploration of novel drug targets in epilepsy.

## 2 Materials and methods

### 2.1 Data collection and processing

Two epilepsy datasets (GSE143272 and GSE256068) were downloaded from Gene Expression Omnibus database (GEO, http://www.ncbi.nlm.nih.gov/geo/). The GSE143272 dataset was a whole-blood gene expression profile consisting of 34 drug-naïve epilepsy patients, 57 followed-up patients with differential responses to antiepileptic drug monotherapy, and 50 healthy controls (HCs). Among them, the gene expression profile of 34 drug-naïve epilepsy patients and 50 HCs in GSE143272 were utilized as a training dataset to screen for ARDEGs signatures. The gene expression profile of 57 followed-up patients with differential response to antiepileptic drugs (valproate, carbamazepine and phenytoin) monotherapy in GSE143272 was employed to verify the characteristics of ARDEGs in the sensitivity of antiepileptic drug monotherapy. In addition, the gene expression profile of 59 temporal lobe epilepsy with hippocampal sclerosis and 11 HC hippocampus samples from GSE256068 were used as independent validation data. Eight hundred three ARGs were obtained from two autophagy-related gene databases HADb^1^ and HAMdb^2^ after eliminating duplicate genes (13, 14). The flowchart was shown in Supplementary Figure S1.

### 2.2 Differential expression genes identification between epilepsy patients and HCs

The DEGs between epilepsy and HC samples was analyzed by the “Limma” package in R software. In the GSE143272 dataset, the threshold were set as |log2FoldChange| ≥0.2 and |*p*-value| <0.05. The top 14 most significant differentially expressed DEGs was then shown in a volcanic map by using “Pheatmap” package in R.

### 2.3 Weighted gene co-expression network analysis

To identify gene-sets significantly related with epilepsy, we construct a weighted co-expression network based on the expression profile and clinical data using the R package “WGCNA” (15). To be specific, after eliminating outlier samples, a suitable soft-power threshold β was selected for automatic network construction through the “pickSoftThreshold” in the R package “WGCNA.” Subsequently, the hierarchical clustering tree was constructed for module detection based on the dissimilarity coefficient between genes. The correlation between module features and clinical characteristics was calculated through Pearson’s correlation analysis. The module genes with the most positively correlation and negatively correlation module genes were used for further analysis.

### 2.4 Identification and functional enrichment of ARDEGs signatures in epilepsy

The ARDEGs signatures in epilepsy was identified using a Venn diagram. GO and KEGG pathway enrichment analysis were performed through the “clusterProfiler” package in R. Among them, GO analyses encompassed molecular functions (MF), biological processes (BP), and cellular components (CC) analysis. The false discovery rate (FDR) <0.05 was regarded as a statistical difference.

### 2.5 Protein–protein interaction and transcription factors and miRNA-gene regulatory network construction

The PPI network was constructed through the Search Tool for the Retrieval of Interacting Genes (STRING; http://string-db.org/). The combined score >0.4 was regarded as statistically significant. The miRNA-gene interaction was searched based on the miRTarBase database (version 9.0). Similarly, the TF-gene interaction was searched based on the ENCODE database. TF-gene and miRNA-gene networks were visualized through the NetworkAnalyst database (16).

### 2.6 Selection of hub ARDEGs in epilepsy

The Cytoscape plug-in CytoHubba was utilized to screen hub genes through three different algorithms, including maximal clique centrality (MCC), degree, and edge percolated component (EPC). The overlapped genes between three algorithms were employed for subsequent analysis. Further, LASSO logistic regression analysis was carried out to obtain the hub genes associated with epilepsy through the “glmnet” package in R.

### 2.7 Immune infiltration analysis

To explore the differences in immune cell infiltration between epilepsy and HCs, the Immune Cell Abundance Identifier (ImmuCellAI) was employed in this study. The ImmuCellAI is an online tool for accurately estimating the abundance of 18 T-cell subtypes and six other important immune cells through the RNA sequencings and microarray datasets (17). The association between hub genes and immune cell abundance was performed by using Spearman’s correlation analysis.

### 2.8 Statistics

In this study, the statistical analyses and graphs were accomplished using GraphPad Prism 9.0 software, R software (version 4.1.2) and SangerBox online analysis tool.^3^

## 3 Result

### 3.1 Integration of differential gene analysis and WGCNA screens out ARDEGs in epilepsy

**Figure 1:**
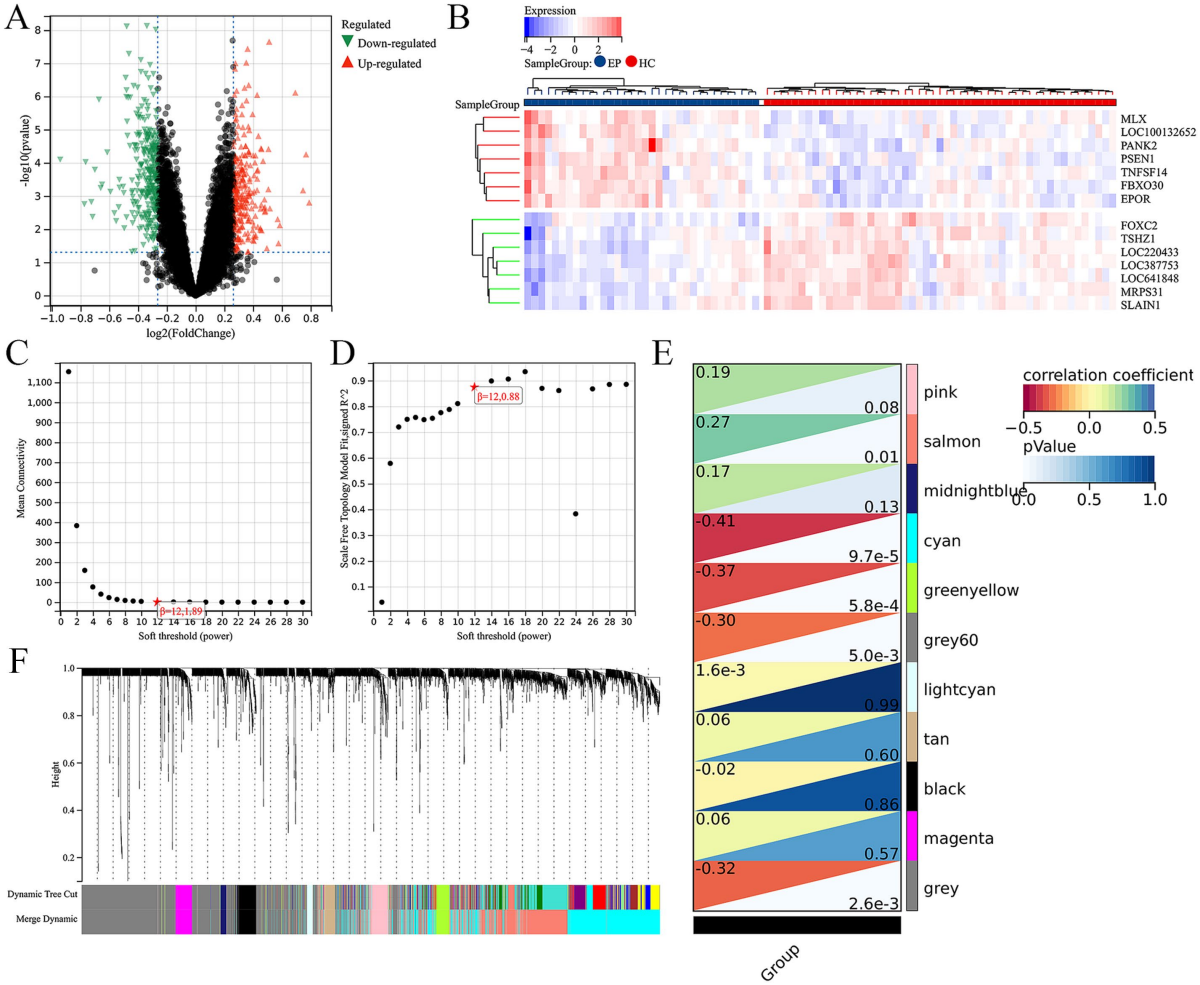
Integration of differential gene analysis and WGCNA screen out ARDEGs in epilepsy. **(A)** Volcanic map of DEGs between the epilepsy and HC. *p*-value <0.05 and |log2FoldChange| ≥0.2. **(B)** The heat map shows the top 14 DEGs between the epilepsy and healthy control. **(C)** The scale-free index for various soft-threshold powers (β). **(D)** The mean connectivity for various soft-threshold powers. **(E)** Eleven recognition module. **(F)** Correlation heat map of phenotypes and gene modules.

**Figure 2:**
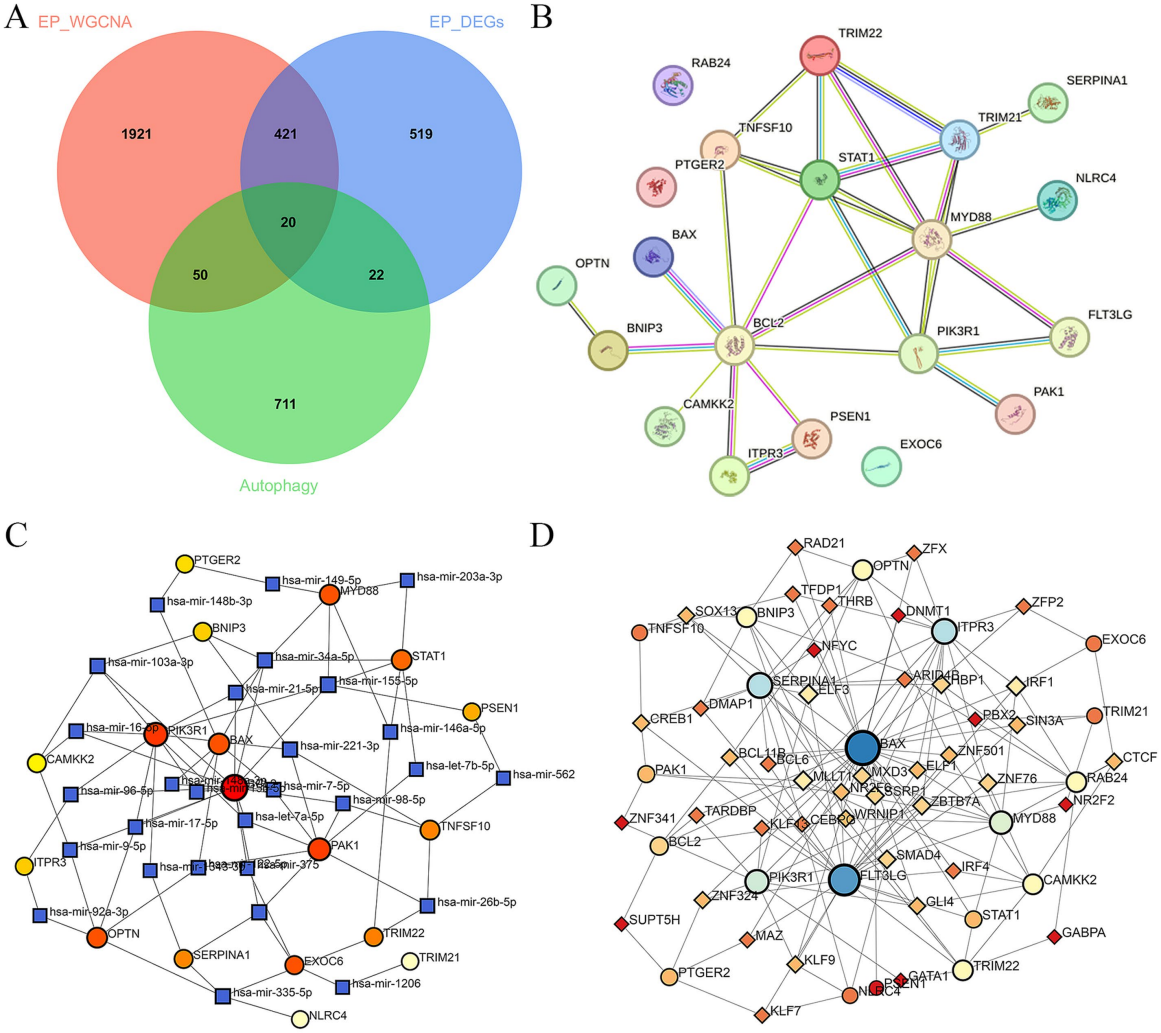
Construction of PPI, TFs, and miRNAs network of ARDEGs in epilepsy. **(A)** The Venn diagram represents the common intersection of DEGs, WGCNA, and ARDEGs. **(B)** PPI network of ARDEGs in epilepsy. **(C)** miRNA-hub cross-talk genes regulatory network. **(D)** TFs-hub cross-talk genes regulatory network.

First of all, we screened for DEGs between epilepsy and HCs with the threshold of |log2FoldChange| ≥0.2 and |*p*-value| <0.05. Total 515 up-regulated and 467 down-regulated DEGs between epilepsy and HCs were identified (Figure 1A). The top 14 DEGs between epilepsy and HCs were presented in the heatmap (Figure 1B). Furthermore, we explored the epilepsy-related genes through WGCNA analysis (Figures 1C,D). The cluster dendrogram classified all genes into 11 modules (Figure 1F). Among them, the salmon module (including 999 genes) was most positively correlated with epilepsy and the cyan module (including 1,413 genes) was most negatively correlated with epilepsy (Figure 1E). Finally, 20 ARDEGs in epilepsy were screened out though integrating DEGs, WGCNA, and ARGs from HADb and HAMdb database (Figure 2A).

### 3.2 Construction of PPI, TFs and miRNAs network of ARDEGs in epilepsy

A PPI network of 20 ARDEGs in epilepsy was showed in Figure 2B. To better understand of the role of ARDEGs in the pathological mechanism of epilepsy, the construction of TF-gene and gene-miRNA networks were carried out in sequence, with a focus on identifying regulatory factors affecting the ARDEGs at both transcriptional and post-transcriptional levels. Using the miRTarBase database, the miRNAs that interact with 20 ARDEGs in epilepsy was identified. Through this analysis, a gene-miRNA network consisting of 46 nodes and 81 edges was constructed (Figure 2C). Additionally, the TFs that could regulate the 20 ARDEGs were predicted using the TRUST database, and a TF-gene regulation network consisting of 63 nodes and 160 edges was constructed (Figure 2D). Our results provide novel insights into the regulatory mechanisms of ARDEGs in epilepsy.

### 3.3 Functional enrichment of ARDEGs in epilepsy

**Figure 3:**
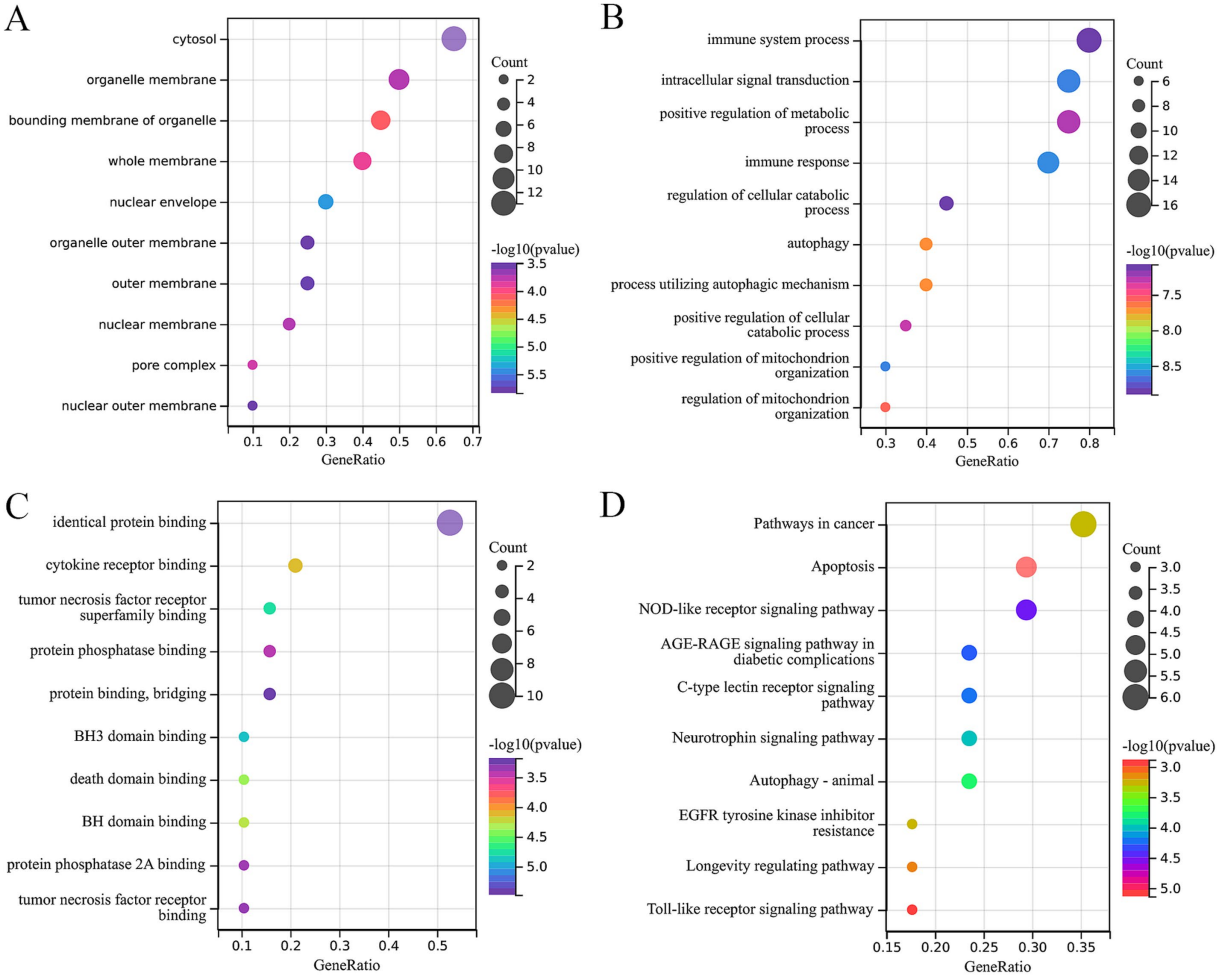
Functional enrichment of ARDEGs in epilepsy. **(A–C)** GO enrichment analysis (cellular components, biological process, and molecular function) of ARDEGs in epilepsy. **(D)** KEGG enrichment analysis of ARDEGs in epilepsy.

To further elucidate the molecular mechanism of ARDEGs in epilepsy, the KEGG and GO enrichment analysis were conducted. The CC analysis indicated that the ARDEGs in epilepsy were predominantly located in cytosol, organelle membrane, bounding membrane of organelle, whole membrane, nuclear envelope, etc. (Figure 3A). Their biological processes were related with immune system process, intracellular signal transduction, positive regulation of metabolic process, regulation of cellular catabolic process, etc. (Figure 3B). The molecular functions of the ARDEGs in epilepsy were implicated in identical protein binding, cytokine receptor binding, tumor necrosis factor receptor superfamily binding, protein phosphatase binding, etc. (Figure 3C). The KEGG enrichment analysis revealed that the ARDEGs in epilepsy were involved in the pathways in cancer, apoptosis, NOD-like receptor signaling pathway, AGE-RAGE signaling pathway in diabetic complications, C-type lectin receptor signaling pathway, neurotrophin signaling pathway, autophagy-animal, etc. (Figure 3D).

### 3.4 Screening hub ARDEGs in epilepsy

**Figure 4:**
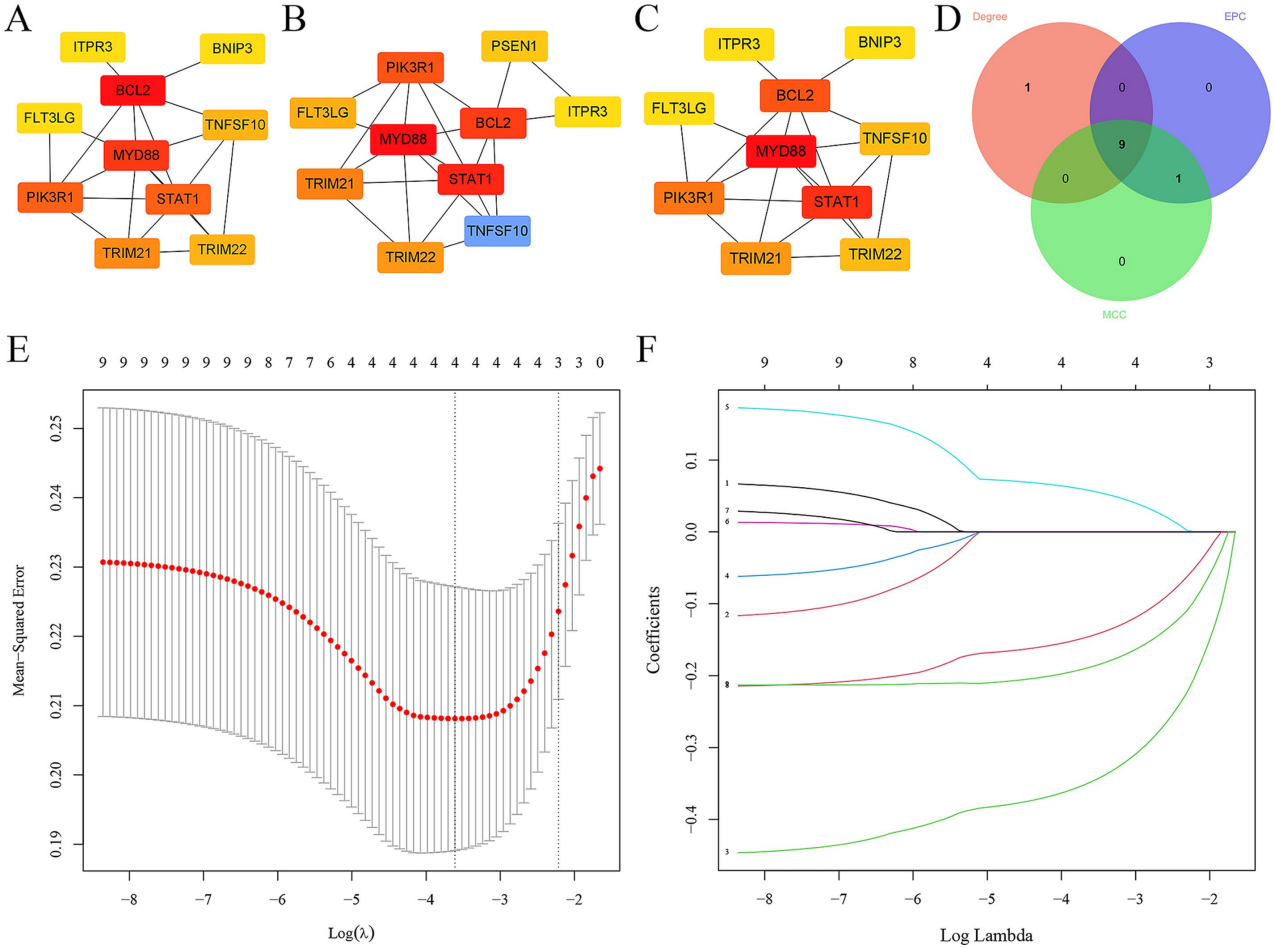
Screening hub ARDEGs in epilepsy. **(A–D)** The top 10 hub ARDEGs were screened by the MNC, MCC, degree, and EPC algorithms of the CytoHubba plugin. **(D)** Venn diagram showing the common hub ARDEGs from three algorithms. **(E)** LASSO coefficient profiles were generated for the initial nine genes that met the prognostic criteria. **(F)** The misclassification error when analyzing the jackknife rate.

In addition, the top 10 hub ARDEGs in epilepsy were screened via CytoHubba plugin in Cytoscape. Three distinct algorithms were employed, including MCC, degree, and EPC (Figures 4A–C). Finally, 9 overlapping hub cross-talk genes between epilepsy and COVID-19 were screened out from three algorithms, including FLT3LG, PIK3R1, BCL2, ITPR3, MYD88, STAT1, TRIM21, TRIM22, and TNFSF10 (Figure 4D). Subsequently, the expression profile of hub ARDEGs was then used to establish the LASSO logistic regression analysis (Figure 4E). Ultimately, four hub ARDEGs were retained, including PIK3R1, TRIM21, TRIM22, and ITPR3 (Figure 4F).

### 3.5 Immune infiltration analysis in epilepsy

**Figure 5:**
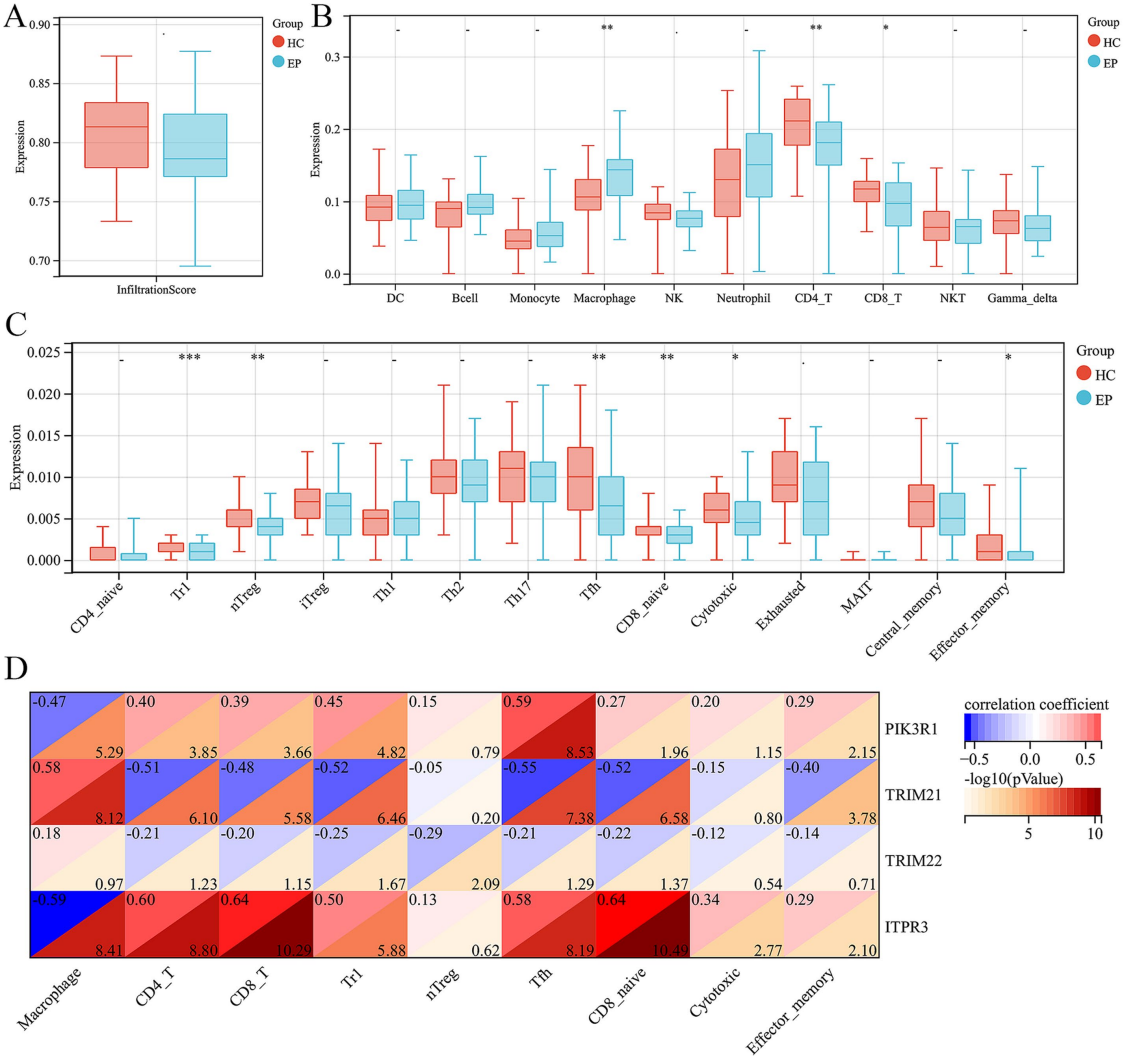
Immune infiltration analysis of epilepsy and their correlation with ARDEGs expression level. **(A)** Infiltration score difference between epilepsy and HCs. **(B)** Ten types of immune cell difference between epilepsy and HCs. **(C)** Fourteen CD4 and CD8T cell subtypes difference between epilepsy and HCs. (D) Correlation analysis between hub ARDEGs and different immune cell abundance in epilepsy. ^*^p < 0.05, ^**^p < 0.01, ^***^p < 0.001, and ^****^p < 0.0001; ns, no statistical difference.

We further employed the ImmuCellAI tool to evaluate the disparity in the abundance of immune cells in epilepsy. First of all, we did not observe any difference in infiltration score between epilepsy and HC group (Figure 5A). In addition, among the 10 immune cell subtypes, there was a significantly increased abundance of macrophage and a decreased abundance of CD4 and CD8 T cell in epilepsy group, compared with HC group (Figure 5B). In the T cell subtype, we discovered a decreased Tr1, nTreg, Tfh, CD8 naïve, cytotoxic T cell and effector memory T cell in epilepsy patient (Figure 5C). We further investigated the potential correlation between hub ARDEGs and the abundance of different immune cells by using Spearman’s correlation analysis. The result showed that the expression level of PIK3R1 was negatively related with the macrophage (*r* = −0.47, *p* < 0.001) and positively related with CD4 T cell (*r* = 0.40, *p* < 0.001), CD8 T cell (*r* = 0.39, *p* < 0.001), Tr1 (*r* = 0.45, *p* < 0.001), Tfh (*r* = 0.59, *p* < 0.001), CD8 naïve (*r* = 0.27, *p* = 0.011) and effector memory T cell (*r* = 0.29, *p* = 0.007) (Figure 5D). The expression level of TRIM21 was positively related with the macrophage (*r* = 0.58, *p* < 0.001) and negatively related with CD4 T cell (*r* = −0.51, *p* < 0.001), CD8 T cell (*r* = −0.48, *p* < 0.001), Tr1 (*r* = −0.52, *p* < 0.001), Tfh (*r* = −0.55, *p* < 0.001), CD8 naïve (*r* = −0.52, *p* < 0.001) and effector memory T cell (*r* = −0.40, *p* < 0.001) (Figure 5D). The expression level of TRIM22 was negatively related with Tr1 (*r* = −0.25, *p* = 0.021) and nTreg (*r* = −0.29, *p* = 0.008) (Figure 5D). The expression level of ITPR3 was negatively related with the macrophage (*r* = −0.59, *p* < 0.001) and positively related with CD4 T cell (*r* = 0.60, *p* < 0.001), CD8 T cell (*r* = 0.64, *p* < 0.001), Tr1 (*r* = 0.50, *p* < 0.001), Tfh (*r* = 0.58, *p* < 0.001), CD8 naïve (*r* = 0.64, *p* < 0.001), cytotoxic T cell (*r* = 0.34, *p* = 0.002) and effector memory T cell (*r* = 0.29, *p* = 0.007) (Figure 5D). Overall, macrophages, CD4 and CD8 T cell subtypes were the immune cells that underwent the most significant abundance changes in epilepsy and were significantly correlated with the expression level of hub ARDEGs.

### 3.6 Validation of hub ARDEGs expression level in antiepileptic drug monotherapy patients and hippocampus of epilepsy

**Figure 6:**
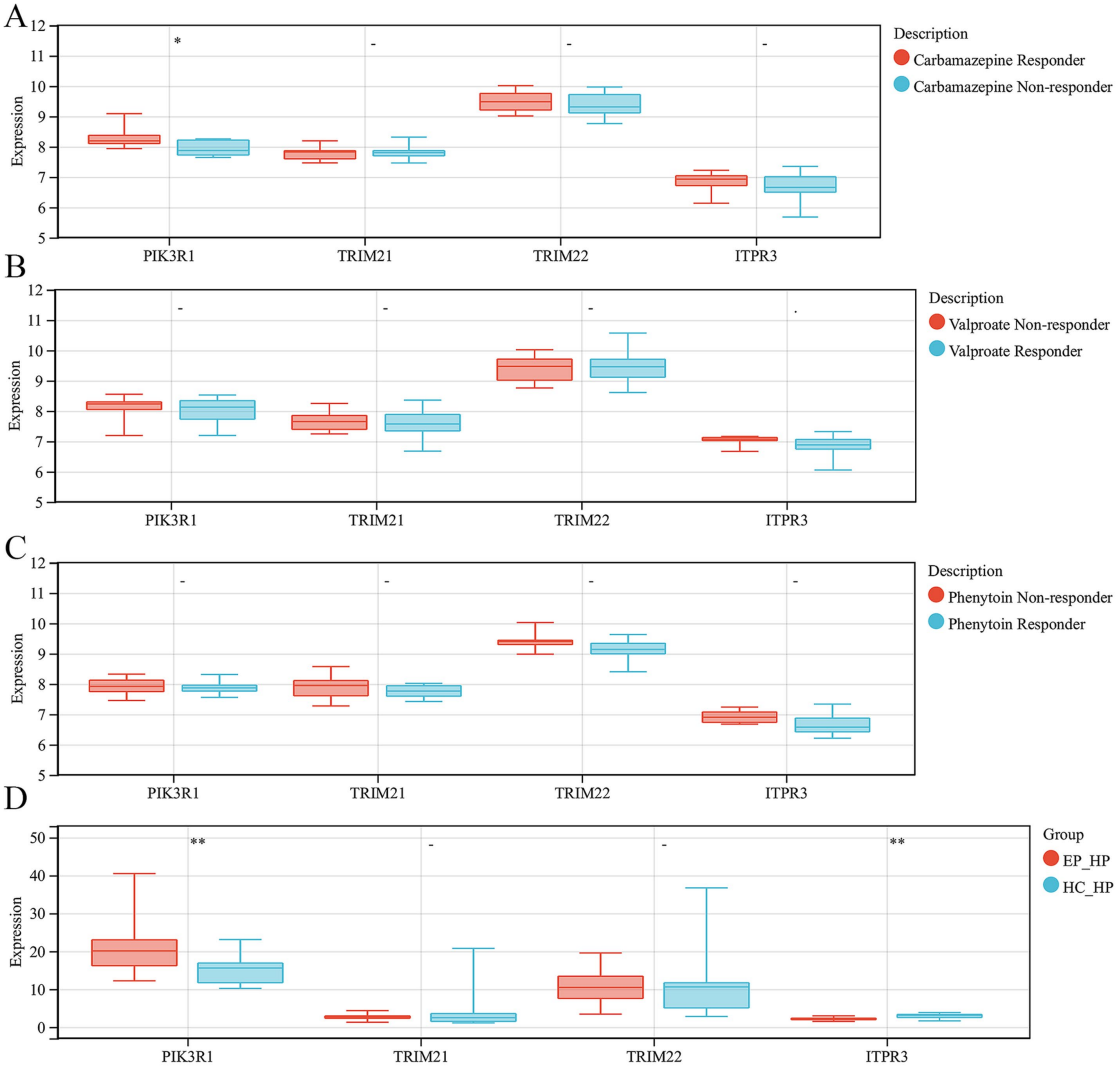
Validation of hub ARDEGs expression level in antiepileptic drug monotherapy patients and hippocampus of epilepsy. **(A)** The difference analysis of ARDEGs expression level between carbamazepine responder and non-responder. **(B)** The difference analysis of ARDEGs expression level between valproate responder and non-responder. **(C)** The difference analysis of ARDEGs expression level between phenytoin responder and non-responder. **(D)** The difference analysis of ARDEGs expression level in the hippocampus of epilepsy and HCs. ^*^p < 0.05 and ^**^p < 0.01.

In order to explore the role of ARDEGs signatures in the response to anti-epilepsy drug treatment, we conducted further analyses the expression levels of hub ARDEGs in patients with different responses to three anti-epilepsy drugs (valproate, carbamazepine and phenytoin). We discovered that there were significant differences in the expression levels of PIK3R1 between carbamazepine responder and non-responder patients. However, there were no significant differences in the expression levels of the remaining hub ARDEGs in valproate and phenytoin treatment (Figures 6A–C). In addition, to verify the consistency of the expression level of hub ARDEGs in epileptic brain tissues, we selected epileptic and HC hippocampal tissue for expression level verification. The results showed that the expression level of PIK3R1 was significantly increased in the hippocampus of epileptic patients, while the expression level of ITPR3 was significantly decreased (Figure 6D). Therefore, PIK3R1 and ITPR3 had significant differences in peripheral blood and brain tissue of patients with epilepsy, and had potential as biomarkers in epilepsy.

## 4 Discussion

Epilepsy, a common neurological disorder, is marked by susceptibility to recurrent seizures (18, 19). The complex pathological processes and distinct individual differences in epilepsy present considerable challenges for clinical intervention. Autophagy is known to be vital in maintaining the balance of the CNS. Nevertheless, there remains an incomplete understanding of the molecular mechanisms related to autophagy in epilepsy, which hinders the advancement of new treatment approaches. Therefore, it is crucial to gain a deeper understanding of the pathophysiology of epilepsy and identify key molecular mechanisms for the development of effective therapeutic strategies.

In this study, we endeavored to discover the autophagy-related gene signatures in epilepsy by integrating bioinformatics methods. First of all, we screened 20 ARDEGs in epilepsy through the integration of DEGs, WGCNA analysis and ARDEGs from HADb and HAMdb database. To obtain a deeper comprehension of the role of ARDEGs in the pathological mechanism of epilepsy, the construction of PPI, TF-gene and gene-miRNA networks were carried out successively, with a focus on identifying regulatory factors influencing the ARDEGs at both transcriptional and post-transcriptional levels. The KEGG enrichment analysis showed that the ARDEGs in epilepsy were not only implicated in the autophagy, but also the pathways in cancer, apoptosis, NOD-like receptor signaling pathway, AGE-RAGE signaling pathway in diabetic complications, C-type lectin receptor signaling pathway, neurotrophin signaling pathway, etc. Recently, research has been conducted to explore the role of autophagy in epilepsy. It has been suggested that autophagy governs the balance between inhibitory GABA and excitatory glutamate, potentially hindering the initiation and progression of epilepsy (10). Similarly, preclinical and clinical research results suggest that drugs targeting the autophagy pathway play an important role in anti-epilepsy effects. For example, rapamycin, an inducer of autophagy, has shown effectiveness in combating epileptogenesis and epilepsy during both the acute and chronic stages of kainic acid-induced seizure in mice (20). Notably, the research on the impact of rapamycin on seizures indicates that it can reduce the frequency of seizures and the usage of antiepileptic drugs (21). Overall, targeting autophagy related pathways may provide an important therapeutic strategy for epilepsy.

Furthermore, 4 hub ARDEGs, including PIK3R1, TRIM21, TRIM22, and ITPR3, were screened by integrating CytoHubba plug in Cytoscape and LASSO logistic regression analysis. More significantly, PIK3R1 and ITPR3 were also significantly differentially expressed in the hippocampus of epilepsy group, compared with HC group. Phosphatidylinositol 3-kinase regulatory subunit alpha (PIK3R1) functions as a regulatory subunit for phosphoinositide 3-kinases (PI3Ks) (22). PIK3R1 is highly present in various tissues such as the brain, liver, muscle, adipose tissue, and kidney, and is considered as an autophagy regulatory factor (22, 23). The PIK3R1/AKT/mTOR signaling pathway plays a role in regulating autophagy level (24). Similarly, this pathway has been extensively studied in pathogenesis of epilepsy (25). In a macaque model of mesial temporal lobe epilepsy, high-frequency hippocampus deep brain stimulation (DBS) can regulate abnormal gene expression levels including PIK3R1 (26). In this study, the expression level of PIK3R1 was found to be associated with carbamazepine resistance. Based on the aforementioned results, we hypothesized that PIK3R1-mediated autophagy was crucial for participating in the development and progression of epilepsy, and could become a new target for the development of anti-epileptic drugs in the future. Inositol 1,4,5 trisphosphate receptors (ITPRs) are a group of Ca^2+^ channels located in the endoplasmic reticulum which play a crucial role in regulating cellular calcium ion homeostasis in all types of mammalian cells (27–29). ITPR3, an isoform of the ITPR family, plays a crucial role in the pathogenesis of various diseases, such as alcoholic hepatitis, breast cancer, glioblastoma, gastric cancer (30). In the peripheral nervous system, it has been proven that dominant mutations in ITPR3 can lead to the onset of Charcot–Marie–Tooth disease, demyelinating, 1J (CMT1J) (28). This study was the first to determine that ITPR3 showed differential expression in the peripheral blood and hippocampus of epilepsy patients, which might play a role in regulating the level of autophagy by modulating neuronal calcium homeostasis.

Numerous studies have indicated that immune cells play a crucial role in promoting the epileptogenesis and progression of epilepsy. To provide greater clarity regarding the connection between autophagy and the immune response, we further utilized the ImmuCellAI tool to evaluate the disparity in the abundance of immune cells in epilepsy. The result indicated that there was a significantly increased abundance of macrophages and a decreased abundance of CD4 and CD8 T cell in epilepsy group, compared with HC group among the 10 immune cell subtypes. In the T cell subtype, we discovered a decreased Tr1, nTreg, Tfh, CD8 naïve, cytotoxic T cell and effector memory T cell in epilepsy patient. In recent years, the role of the immune system in epilepsy has attracted much attention (31). Mounting evidence have showed that glia activation accompanied by the biosynthesis and release of the inflammatory factor in animal models of epilepsy (32–34). In addition, an increased number of peripheral immune cells, such as macrophages and neurotrophils, have also been reported in the brain of epilepsy during epileptogenesis (35, 36). Pervious study has showed that epilepsy patients had a relative increase of total leukocytes, neutrophil leukocytes, total lymphocytes, NK cells, epinephrine, and decreased CD4^+^ T cells in the immediate postictal state (37). The results reported in the previous studies were consistent with the immune cell abundance changes observed in this study.

In addition, it was discovered in this study that the expression level of hub ARDEGs was significantly correlated with the abundance of differential immune cells. The level of PIK3R1 expression showed negative correlation with macrophages and positive correlation with CD4 T cells, CD8 T cells, Tr1, Tfh, CD8 naive and effector memory T cells. The level of TRIM21 expression was positively associated with macrophages and negatively associated with CD4 T cells, CD8 T cells, Tr1, Tfh, CD8 naive and effector memory T cells. The level of TRIM22 expression was negatively correlated with Tr1 and nTreg. The level of ITPR3 expression was negatively related to macrophages and positively related to CD4 T cells, CD8 T cells, Tr1, Tfh, CD8 naive, cytotoxic T cell and effector memory T cell.

There were several limitations in this study. The results were established on the GEO database, and they have not been validated *in vivo* or *in vitro*. Secondly, the sample size of this study was relatively small, and including more patients would be beneficial for enhancing the general applicability of our findings. In addition, we did not delve into the differences in innate immune cells such as M1 and M2 macrophages in epilepsy, nor the differences in the abundance of peripheral immune cells infiltrating the epilepsy brain tissue. Finally, the functions of the hub ARDEGs identified in this study were still unclear in epilepsy and need to be further explored in future studies. Revealing these findings may contribute to the discovery of new treatment targets and approaches for managing epilepsy.

## 5 Conclusion

This study elucidates the autophagy-related gene signatures (PIK3R1, TRIM21, TRIM22, and ITPR3) in epilepsy and clarifies their association with immune infiltration and their role in anti-epileptic drug responsiveness, which provide a novel target for future therapeutic interventions and disease markers in epilepsy.

## Data Availability

The original contributions presented in the study are included in the article/Supplementary material, further inquiries can be directed to the corresponding author.

## Footnotes

^1^ http://www.autophagy.lu/index.html

^2^ http://hamdb.scbdd.com/

^3^ http://vip.sangerbox.com/home.html

## Supplementary material

SUPPLEMENTARY FIGURE S1 The flowchart of this study.
