# Supplementary figures and images for: Identification and verification of autophagy-related gene signatures and their association with immune infiltration and drug responsiveness in epilepsy
Source: Front Neurol. 2025 Jan 22;15:1503632. doi: 10.3389/fneur.2024.1503632 (PMC11794110; doi:10.3389/fneur.2024.1503632)

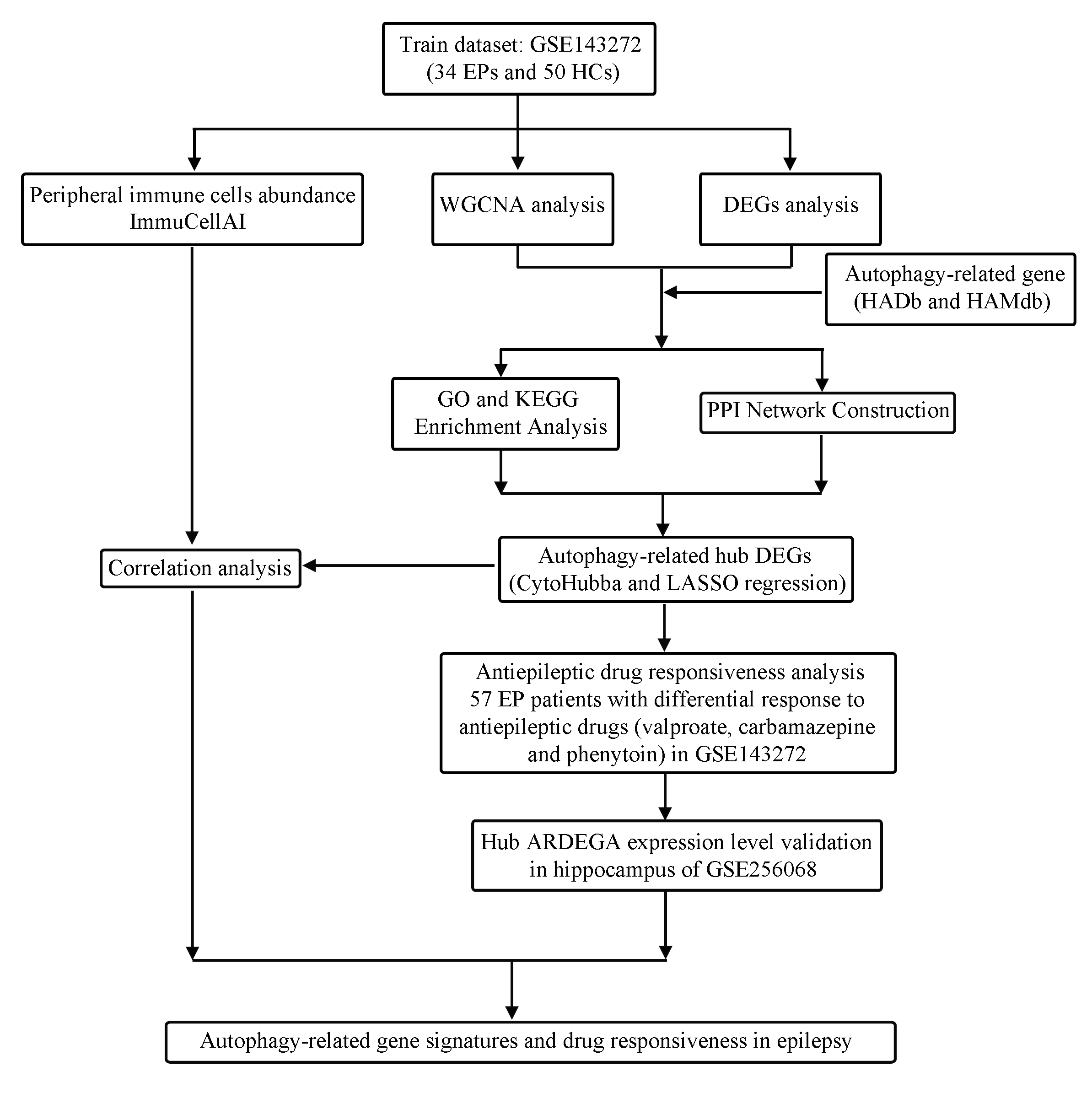

Supplement: SUPPLEMENTARY FIGURE S1 — The flowchart of this study. [file Image_1.tif]
